# Pembrolizumab Induces an Unexpected Conformational Change in the CC′-loop of PD-1

**DOI:** 10.3390/cancers13010005

**Published:** 2020-12-22

**Authors:** Bernhard Roither, Chris Oostenbrink, Georg Pfeiler, Heinz Koelbl, Wolfgang Schreiner

**Affiliations:** 1 Institute of Biosimulation and Bioinformatics, Medical University of Vienna, Spitalgasse 23/88.04, 1090 Vienna, Austria; bernhard.roither@meduniwien.ac.at; 2 Institute of Molecular Modeling and Simulation, University of Natural Resources and Life Science, Muthgasse 18, 1190 Vienna, Austria; chris.oostenbrink@boku.ac.at; 3 Department of Obstetrics and Gynecology (Division of General Gynecology and Gynecologic Oncology), Medical University of Vienna, Waehringer Guertel 18-20, 1090 Vienna, Austria; georg.pfeiler@meduniwien.ac.at (G.P.); heinz.koelbl@meduniwien.ac.at (H.K.)

**Keywords:** immunotherapy, checkpoint inhibitors, molecular dynamics simulation, molecular conformation, antibodies

## Abstract

**Simple Summary:**

Cancer cells are normally destructed by killer T-cells. However, T-cells expose the PD-1 receptor on their surface, acting as a checkpoint: If it is activated through a special molecule, PD-L1, the T-cell kills itself, ending the attack. Cells often need to present PD-L1 to prevent T-cells from over-aggressive attacks which cause autoimmune disease. There are tumors which also present PD-L1, thereby evading natural clearing, allowing them to continue growing. New anticancer drugs (checkpoint inhibitors: nivolumab and pembrolizumab) disrupt this evasion: They competitively bind to PD-1, without activating it, and re-enable immune tumor destruction. We scrutinize the binding mechanisms via molecular dynamics simulation. We demonstrate that these drugs deform the CC′-loop of the PD-1 in ways differing from those seen with PD-L1 as a binding partner. Pembrolizumab induces a new conformation of the CC′-loop not known to date. These findings might pave the way for the development of new anti-cancer drugs.

**Abstract:**

To improve cancer immunotherapy, a clearer understanding of key targets such as the immune checkpoint receptor PD-1 is essential. The PD-1 inhibitors nivolumab and pembrolizumab were recently approved by the FDA. The CC′-loop of PD-1 has been identified as a hotspot for drug targeting. Here, we investigate the influence of nivolumab and pembrolizumab on the molecular motion of the CC′-loop of PD-1. We performed molecular dynamics simulations on the complete extracellular domain of PD-1, in complex with PD-L1, and the blocking antibodies nivolumab and pembrolizumab. Conformations of the CC′-loop were analyzed unsupervised with the Daura et al. clustering algorithm and multidimensional scaling. Surprisingly, two conformations found were seen to correspond to the ‘open’ and ‘closed’ conformation of CC′-loop in apo-PD-1, already known from literature. Unsupervised clustering also surprisingly reproduced the natural ligand, PD-L1, exclusively stabilizing the ‘closed’ conformation, as also known from literature. Nivolumab, like PD-L1, was found to shift the equilibrium towards the ‘closed’ conformation, in accordance with the conformational selection model. Pembrolizumab, on the other hand, induced a third conformation of the CC′-loop which has not been described to date: Relative to the conformation ‘open’ the, CC′-loop turned 180° to form a new conformation which we called ‘overturned’. We show that the combination of clustering and multidimensional scaling is a fast, easy, and powerful method in analyzing structural changes in proteins. Possible refined antibodies or new small molecular compounds could utilize the flexibility of the CC′-loop to improve immunotherapy.

## 1. Introduction

### 1.1. General

Emerging cancer cells are normally recognized by T-cells [1] and destructed. However, escaping the immune-mediated destruction is a hallmark of cancer cells [2], accomplished by abusively activating immune checkpoint molecules [3]. Altering their expression and/or the release of immunosuppressive factors can help the tumor survive even in a ‘hot’, immune cell enriched surrounding.

The immune checkpoint receptor programmed cell death protein 1 (PD-1) belongs to the group of secondary co-inhibitory receptors. Its natural ligand, PD-L1, can be expressed by tumor cells and can interact with PD-1 as well as with B 7.1 on the T-cell, thereby downregulating T-cell activity. The engagement of PD-1 with PD-L1 disrupts the vital T cell receptor (TCR) signals which results in the termination of cytokine production, halts the cell cycle, and ultimately causes apoptosis of antigen-specific T cells [4]. Blocking PD-L1 or PD-1 may therefore be a targeted anti-cancer therapy [5,6]. Delivery of blocking antibodies via nanoparticles is a field of intense research [7,8].

Because of PD-L1s ubiquitous expression PD-1 is also a crucial factor in preventing autoimmunity. The immunosuppressive effect of the PD-1 pathway is exploited by various cancer types that sometimes elude the immune system.

### 1.2. Clinical Significance

Clinical trials in various cancer types have been launched in an attempt to prove efficacy of this strategy [9]. In breast cancer, for example, clinical Phase I and II trials showed mixed results regarding antibodies directed against PD-L1, such as atezolizumab, and against PD-1, such as pembrolizumab or nivolumab [10]. More recently, a phase III trial investigating atezolizumab in metastastic triple negative breast cancer patients showed a distinct improvement in progression-free survival and overall survival, especially in patients with PD-L1 expression [11]. This demonstrates the significance of the target (PD-1) being expressed when a PD-L1 antibody is used. However, no data in breast cancer research exists to clarify whether the expression of PD-1 is predictive when PD-1 checkpoint inhibitors, such as pembrolizumab, are used.

More recently, the application of the PD-1 inhibitors nivolumab and pembrolizumab was permitted in various cancer types. Both monoclonal antibodies bind to PD-1 and block the interaction with PD-L1. In the KEYNOTE-119 study, patients with pretreated metastatic triple negative breast cancer (TNBC) were randomized to receive single pembrolizumab versus a physician’s choice of chemotherapy [12]. No overall survival benefit could be reported in the intention-to-treat (ITT) population. Interestingly, with an increase of PD-L1 expression, a trend for improved survival of patients treated with pembrolizumab was seen. However, data on PD-1 are lacking. In KEYNOTE-522, Schmid et al. demonstrated a significantly higher pathologic complete response (pCR) as a surrogate parameter for disease outcome in TNBC patients treated with neoadjuvant chemotherapy plus pembrolizumab rather than chemotherapy plus placebo [13]. PD-L1 expression did not impact the efficacy of pembrolizumab. Again, data on PD-1 are lacking. The systemic administration of the blocking antibodies can be accompanied by severe side effects. Reducing the side effects and improving the efficacy of the immunotherapy is a Herculean task we must face. In order to do so, a better understanding of the molecular mechanism of the PD-1 receptor is necessary.

### 1.3. Molecular Mechanisms

**Figure 1:**
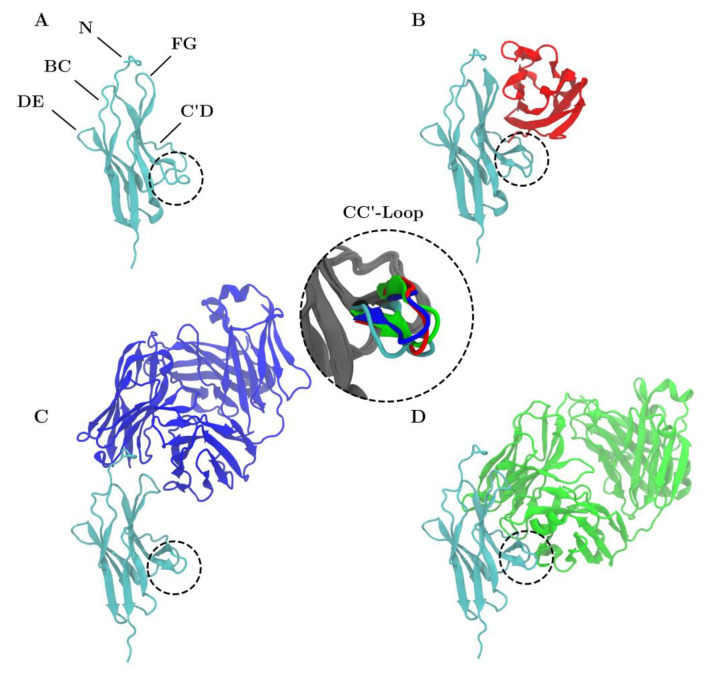
Structure of PD-1 (cyan) is shown unbound (**A**) and in complex with PD-L1 (**B**), nivolumab (**C**) and pembrolizumab (**D**). The CC′-loop is highlighted with a dashed circle. The color of the CC′-loop in the inset denotes the system of origin. The CC′-loop of PD-1_Apo_ is in the open configuration whereas the CC′-loop of PD-1_PD-L1_ (red), PD-1_Niv_ (blue), and PD-1_Pem_ (green) are in the ‘closed’ configuration.

Generally speaking, the CC′-loops of immune checkpoint receptors are crucial for ligand interaction and the CC′-loop of PD-1 is no exception [14]. In X-ray crystallography experiments a shift from the ‘open’ to ‘closed’ conformations of the CC′-loop upon PD-L1 binding was identified [15], see the different colored loops on the enlarged insert within Figure 1. Molecular dynamics (MD) simulations, however, showed that PD-1 in the unbound state exhibits both the ‘open’ and the ‘closed’ conformations and that PD-L1 accelerates the ‘open’-to-‘closed’ switching and eventually locks the ‘closed’ conformation [16]. X-ray crystallography experiments showed an unexpected involvement of the N-loop of PD-1 in the binding process with nivolumab [17]. The fact that it was only possible to crystallize the N-loop with nivolumab underlines the necessity of antibody related research to obtain a better understanding of PD-1. The lack of complete PD-1 crystal structures slows down the development of small molecule inhibitors [18,19]. Hence, through in silico preprocessing we completed the PD-1 crystal structure which will help gain insight into the CC′-loop movements mimicking the natural behavior. Here, we present the results of MD simulations of a complete extracellular domain of PD-1 in complex with PD-L1 and the two clinically used blocking antibodies nivolumab and pembrolizumab.

## 2. Material and Methods

An approach similar to our previous work was taken [20] using the notation PD-1_Apo_, PD-1_PD-L1_, PD-1_Niv,_ and PD-1_Pem_ when referring to the respective simulated system (PD-1 unbound, the PD-1/PD-L1 complex, the PD-1/nivolumab complex and the PD-1/pembrolizumab complex).

### 2.1. Preprocessing

The crystal structures PD-1 unbound (3RRQ), PD-1/PD-L1 (4ZQK), PD-1/nivolumab (5WT9), and PD-1/pembrolizumab (5GGS) were used for MD simulations. The C’D- (residues 85–92) and N-loop (25-34) were missing in 3RRQ and 4ZQK and were taken from 5GGS and 5WT9, respectively, and were added to the other crystal structures. PD-1s of each crystal structure were aligned using the software VMD 1.3 [21] and the missing loops were copied into the PDB files. Hence, each system included the complete extracellular domain of PD-1 (residues 25 to 149).

### 2.2. Simulation

MD simulations were run on the Vienna Scientific Cluster (VSC) using the GROMACS 2018.1 software package [22,23] and the GROMOS 54A7 force field [24]. Protonation states were selected according to neutral pH with deprotonated Asp and Glu, protonated Lys, and Arg residues. His residues were simulated in their neutral state with the proton attached to Nε. This protonation is the standard choice in GROMACS, because it is experimentally known to be the more commonly used one. Molecules were simulated in a cubic SPC water box under periodic boundary conditions with a minimum distance of 1.0 nm to the boundary. The proteins were solvated in SPC water [25] as appropriate for the force field used. An appropriate number of solvent molecules was replaced by Na^+^ and Cl^−^ ions in order to arrive at a physiological salt concentration of 0.15 mol/L. For energy minimization the steepest descent algorithm was applied, with a step size of 0.01 nm and a maximum number of 5 × 10^4^ steps. Minimization was stopped once the maximum force was smaller than 0.1 kJ mol^−1^ nm^−1^. Both, NVT and NPT equilibration were carried out for 0.1 ns with 5 × 10^4^ steps and a step size of 2 fs. Velocity rescaling [26] and the Berendsen barostat [27] were used to keep a constant temperature and pressure of 300 K and 1 bar, respectively. The LINCS algorithm [28] was applied to constrain all bonds to their optimal length. The Verlet cut-off scheme [23] was used for the neighborhood search; the short-range van der Waals cut-off and the long-range electrostatic cut-off were both set at 1.4 nm. Long-range electrostatic forces were summed with Particle Mesh Ewald [29].

After equilibration, each system (PD-1_Apo_, PD-1_PD-L1_, PD-1_Niv_, and PD-1_Pem_) was simulated for 200 ns with a time step of 2 fs and the coordinates were stored every 10 ps. These trajectories entered cluster analysis, as described below. Convergence of the simulations were confirmed, based on clustering (see below). Additionally, for each system, ten independent, shorter simulations of 10 ns length were performed, each starting from different initial velocities. The 10 ns simulations were not used for clustering.

### 2.3. Analysis

The root-mean-square fluctuation (RMSF) of atomic positions is the measure of displacement of an atom **x** from its mean position $\bar{x}$:

(1) $$\mathrm{RMSF}\left(x\right)=\sqrt{\frac{1}{T}\sum_{i=1}^{T}|\left|x\left(t_{i}\right)-\bar{x}\right|{|}^{2}}$$

where *T* is the total number of time steps within the respective trajectory. C_α_ atoms of PD-1 were used for RMSF calculations. Because the RMSF is known to diverge between individual simulations, the 10 sub-simulations of 10 ns each were used to compute the overall RMSF for each system.

The root-mean-square deviation (RSMD) of atomic position is the deviation of a structure at a time *t* from a given reference structure at time *t*_ref_:

(2) $$\mathrm{RMSD}\left(t_{\mathrm{ref}},t\right)=\sqrt{\frac{1}{N}\sum_{i=1}^{N}|\left|x_{i}\left(t\right)-x_{i}\left(t_{\mathrm{ref}}\right)\right|{|}^{2}}\;$$

where **x**(*t*) is the position of atom *i* at time *t* and *N* is the total number of atoms of the structure in question. Here, only C_α_ atoms were considered. Fitting was performed on the framework adjacent to the CC′-loop (i.e., PD-1 without the CC′-loop). The RMSD was calculated for the CC′-loop [30]. Both, RMSF and RMSD were calculated in Matlab R2015b (8.6.0.267246) 64-bit.

One of the key algorithms used for evaluation is the Daura et al. clustering algorithm [31]. If the RMSD between two structures is below the cut-off of 0.15 nm, the structures are considered to belong to the same CC′-loop conformational-cluster. The Daura et al. clustering algorithm consists of the following steps:

1. Adopt each structure as center of separate cluster (central frame).
2. Count number of structures within the cut-off i.e., the number of neighbors.
3. Select center with most neighbors, designate it as a cluster and remove the complete set of structures from the ensemble.
4. Repeat until all structures have been assigned to a cluster.

The second key evaluation method is non-metric MultiDimensional Scaling (MDS), applied to the central frames of the biggest 25 clusters to display the structures in a representative two-dimensional space [32].

Note that both Daura-clustering and MDS are so-called non-supervised techniques. This indicates that they work without any human interventions or intermediate decisions and obtain their results solely on the basis of formal, mathematical procedures. If these procedures are well designed, the results of non-supervised techniques in the end may prove to closely mimic intuitive human inspection, while having the advantage of inter-subjectivity and exact reproducibility.

## 3. Results and Discussion

The aim of this MD study was to identify the influence of the clinically used antibodies nivolumab and pembrolizumab on the CC′-loop of PD-1. The CC′-loops play a vital role in ligand affinity across all immune checkpoint receptors. In 49% of the immune checkpoint receptors the CC′-loop, usually only about five residues long, makes up more than 10% of the buried surface [14]. Also, in PD-1 the CC′-loop, consisting of seven amino acids, contributes 15.22% of the total PD-1/PD-L1 interaction interface.

**Figure 2:**
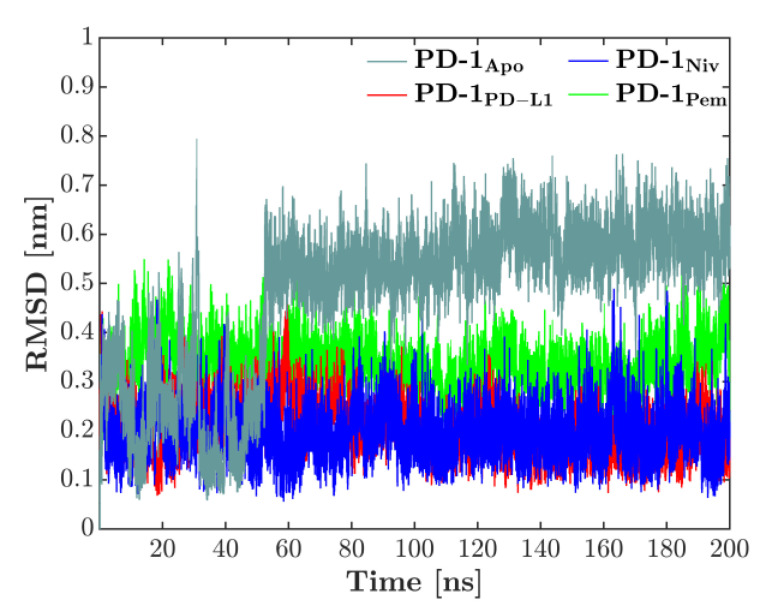
RMSD of CC‘-loop in all 4 systems, computed versus the first configuration of each simulation. While all bound states show regular fluctuations, the unbound system (PD-1_Apo_) exhibits a fluctuating behavior, starting at a lower level around 0.3 nm. Around 50 ns the CC′-loop deforms and accordingly the RMSD increases to 0.6 nm. Note that also around 30 ns, a very short, reversible jump is visible.

The overall deformation of the CC′-loop was characterized by the RMSD, computed against the first configuration of each respective simulation, see Figure 2. The time-series of the RMSD showed that for the complexes, the loop behavior was quite converged for the 200 ns used in the simulations. No more drifts appeared in the RMSD, only sudden jumps for the PD-1_Apo_, indicating skips between two conformations: After starting around 0.3 nm, a sudden increase in RMSD reflected a first but transient deformation of the CC′-loop at 30 ns. A second jump around 50 ns indicated the skip into a configuration which then lasted throughout the remaining simulation (200 ns). Each of the three bound states fluctuated more or less around its respective, constant level: PD-1_PD-L1_ and PD-1_Niv_ around 0.2 nm, PD-1_Pem_ around 0.35 nm. These observations will ultimately lead to a compelling interpretation later in this work.

### 3.1. Local Flexibility

**Figure 3:**
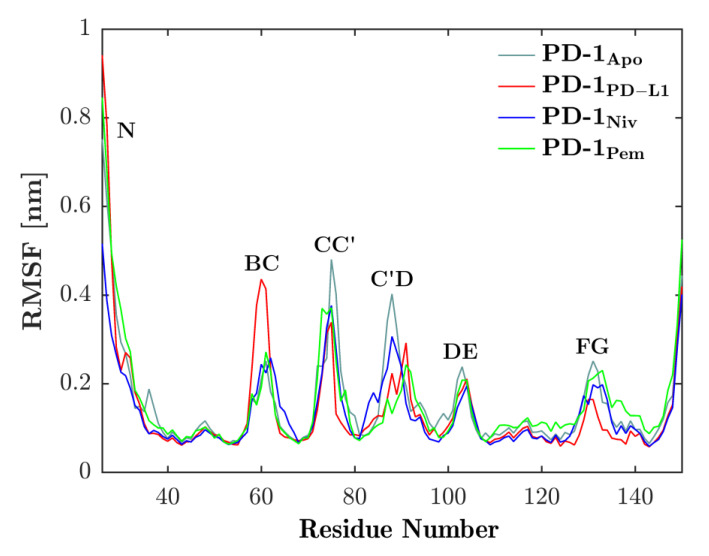
Binding partners reduce flexibility of CC′-loop. RMSF of C_α_s (see residue number on horizontal axis) of PD-1 is shown for binding partners PD-L1 (red), nivolumab (blue) and pembrolizumab (green). Each of the binding partners decreases the flexibility of the CC′-loop in comparison to the unbound state (cyan). Except for the BC-loop, a similar behavior can be seen in the other loops.

We investigated the local motility along the backbone of PD-1 by fitting each frame to the first frame of the respective trajectory and then computing the RMSF of all C_α_s according to Equation (1); see the result in Figure 3. The vital role of the CC′-loop for interfacing with binding partners, as is known from past experiments, was confirmed by a reduced RMSF (i.e., motility) as the binding partners engaged. Similarly, the C’D-loop experienced a similar reduction in motility upon binding. As opposed to this, the BC-loop seemed to increase motility when bound to the natural ligand, PD-L1. The dynamics of the BC-loop has already been evaluated in our previous work [20] where we were able to show that it exhibits three different conformations depending on the binding partner.

#### 3.1.1. Conformations of CC′-Loop

In addition to decreased motility, the formation of distinct conformations makes the CC′-loop pivotal in the binding process. Based on crystal structures, two conformations, ‘open’ and ‘closed’, of the CC′-loop were described [15]. PD-1_Apo_ displayed the loop in an ‘open’ conformation which pointed away from the PD-L1 binding site. Upon complex formation, the CC′-loop performed a 90° turn towards the ligand, allowing the formation of four hydrogen bonds between receptor and ligand. This turn was enabled by the rearrangement of the Ile134 (and Glu136) side chains, making way for the CC′-loop [15]. Zak et al. described that the ‘closed’ CC′ conformation was also found in murine PD-1_Apo_ (but not in human), which led to the hypothesis that the induced CC′-loop rearrangement was specific to human PD-1.

#### 3.1.2. Key Conformations Identified by Clustering

In order to further scrutinize the impact of ligands on the structure of the CC′ loop, we evaluated our simulation results based on conformational clustering. The trajectories of each PD-1 simulation were extracted, concatenated and the RMSD of the CC′-loop was calculated between each pair of configurations. Using these RMSDs as a measure of distance, the Daura et al. clustering algorithm was applied.

First, we assessed the convergence of our simulations by considering the first part (50 ns) of the simulations of our four systems (PD-1_Apo_, PD-1_PD-L1_, PD-1_Niv_, and PD-1_Pem_), concatenating these into a single (4 × 50 ns = 200 ns) trajectory, and subsequent clustering. The number of clusters, *N*_c_, was recorded. Next, larger parts of each trajectory were concatenated, and the procedure was repeated. With increasing length of trajectories, we first observed an increase in *N*_c_, but around 150 ns per system, *N*_c_ levelled off, indicating that more simulation time did not lead to the sampling of additional areas of phase space and that the simulation time of 200 ns per system could be considered sufficient for convergence. Figure S1 shows how the number of clusters first increased with total simulation time and started to level off after about 600 ns (see also [20]). A small continuous increase was to be expected, but the clusters later appearing were typically clusters with only 1–2 observations per cluster.

**Figure 4:**
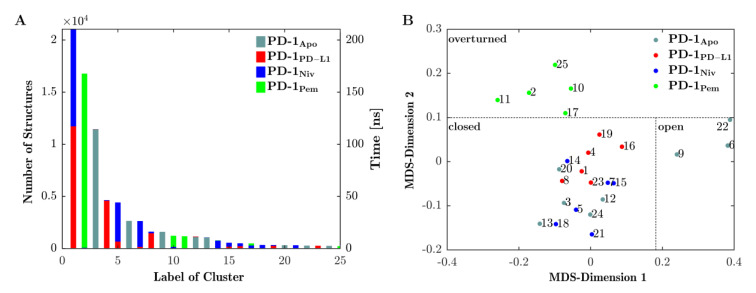
The CC‘-loop exhibits three conformations. (**A**) CC′-loop structures along the 4 joined trajectories (see legend) are clustered (clusters labelled 1–25) and the number of structures belonging to each cluster are displayed on the left vertical axis. The number of clusters is equivalent to the time during which the CC′-loop assumes a configuration within the respective cluster (right vertical axis, time [ns]). Similar structures may originate from one or from more trajectories, hence bars maybe uniquely colored or stacked in color, respectively. For example, the conformation seen in cluster 2 appears only in trajectory PD-1_Pem_ (green) whereas cluster 1 appears in both PD-1_PD-L1_ (red) and PD-1_Niv_ (green). (**B**) The central structures of each cluster were projected into 2-dimensions (MDS-dimensions 1 and 2) by multidimensional scaling (MDS). Central structures are colored according to the trajectory from which they originate, similar to panel (**A**). MDS groups central structures of the clusters according to mutual similarity, allowing for an interpretation of similarity between clusters. Resulting groups can be identified to represent certain types of conformations: ‘open’, ‘closed’ or ‘overturned’, see Figure 5 and Figure 6 for 3D visualizations. Note that the labels ‘open’, ‘closed’, and ‘overturned’ do not directly emerge from meta-clustering but are coined after visual interpretation of central frames, as shown in Figure 5. Nevertheless, for clarity we already use them in this figure. Dotted lines between clusters – delineating the meta-clusters—are drawn by hand.

Figure 4A shows the results of the final clustering of the 4 × 200 ns = 800 ns trajectory of loop dynamics. Since the trajectories of all four systems (PD-1_Apo_, PD-1_PD-L1_, PD-1_Niv_ and PD-1_Pem_) were concatenated in advance, the clustering procedure had no information from which (of the four) given systems a configuration originated. Clustering occurred solely according to similarity (RMSD) of configurations, regardless of the system they originated from. As a consequence, configurations from different systems (origins) could belong to the same cluster (mixed clusters, e.g., clusters 1 originated from PD-1_PD-L1_ (red) and PD-1_Niv_ (blue)). Or else, clusters could consist of configurations from only a single system (homogenous clusters, e.g., cluster 3 originated from PD-1_Apo_ only).

Looking at it in reverse, the emergence of a mixed cluster would mean that similar configurations (of the CC′-loop) occurred in more than one system—these systems ‘shared’ those conformations. Such findings are essential to compare functional similarities and differences of the four investigated systems.

The number of structures within a cluster (left vertical axis in Figure 4, panel (A)) is equivalent to the amount of time the CC′-loop spent in conformations characteristic for that particular cluster (see right vertical axis in panel (A)):

1. PD-1_PD-L1_ (red) and PD-1_Niv_ (blue) exhibit the same CC′-loop conformation (cluster 1) for 110 ns and 100 ns, respectively.
2. Structures of PD-1_Apo_ (cyan) belong to several clusters 3, 6, 9, 12, and 13 for 105 ns, 25 ns, 11 ns, 10 ns, and 9 ns, respectively.
3. PD-1_Pem_ (green) belongs to clusters 2, 10, 11 and (marginally) 17 and 25.
4. Cluster 2 is a homogenous cluster consisting of 16,500 structures of PD-1Pem (green).

In particular, our simulations subjected to Daura et al. clustering [31] revealed that 85% of the structures of PD-1_PD-L1_ exhibited the same conformation of the CC′-loop as 55% and 5% of the structures of PD-1_Niv_ and PD-1_Pem_, respectively (Figure 4, panel A, cluster 1). In contrast, PD-1_Apo_ alternated predominantly between two states (clusters 2 and 5). This provided the explanation for the fluctuating behavior of its RMSD, as shown above in Figure 2. The structures of PD-1_Pem_ were distributed across more clusters (1, 3, 6, 9 and 10), whereas the structures of PD-1_Niv_ appeared mainly in two clusters, namely 1 and 4.

#### 3.1.3. Grouping Clusters According to Similarity in Structure of CC′-loop

In the first step toward grouping, the structural characteristics of each cluster was represented by a so-called ‘central structure’. The central structure was one member (a particular structure) of the respective cluster representing the most characteristic one of all structures within that cluster. Similarities (as well as differences) in structure between clusters could thus be quantified by corresponding similarities (as well as differences) between their central structures: The RMSD between central structures lent itself to a one-dimensional proxy for a multidimensional distance between any two clusters in question.

This concept allowed us to apply the following procedure:

1. Characterize similarity relations between clusters by unsupervised multidimensional scaling (MDS) into a two-dimensional plane.
2. Draw border lines between groups of clusters.
3. Interpret the structures in each resulting group visually in VMD, and finally.
4. Relate structural characteristics found in each group either to findings already described in the literature or report them as novel.

Central structures were subjected to MultiDimensional Scaling (MDS) [32,33], based on their pairwise RMSD-distances. Each central structure was thereby projected onto a point in a 2-dimensional plane, see Figure 4B. Note that values along MDS-axes do not have direct interpretation in terms of measured values in corresponding units. Instead, distances between points in 2 dimensions mimics distances of corresponding central frames in high dimension. Hence, those central structures that represented similar conformations, appeared in proximity upon projection into the 2-dimensional plane. The other way round: Grouping of points in the 2-dimensional plane served as proxy for grouping clusters in multidimensional space. In the following, groups of clusters are called ‘meta-clusters’.

### 3.2. Visual Characterization of Meta-Clusters and Naming

**Figure 5:**
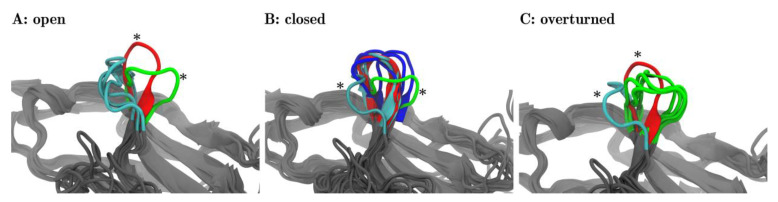
Fleur-de-CC′—Upon pembrolizumab binding the CC′-loop blooms ^1^. The central structures of the 25 clusters are superimposed with VMD in groups as they were meta-clustered in the distance map (Figure 4B)—‘open’ (**A**), ‘closed’ (**B**), and ‘overturned’ (**C**). The central frames are colored according to the origins of the structures: PD-1_Apo_ (cyan), PD-1_PD-L1_ (red), PD-1_Niv_ (blue), and PD-1_Pem_ (green). Each panel focuses on a different meta-cluster, A on ‘open’, B on ‘closed’ and C on ‘overturned’. In each panel, *all* of the central frames are displayed for the respective meta-cluster. Additionally, for the two remaining meta-clusters, *one* representative frame is shown as a comparison (marked with an asterisk *). For example: panel A (‘open’) shows the three central structures (cyan) originating from PD-1_Apo_, all within meta-cluster ‘open’, cf. the three structures (6, 9, 22) in cyan, labelled ‘open’ in Figure 4. In addition, panel (**A**) shows one representative structure of meta-cluster ‘closed’ (in focus in panel B) and one central frame of meta-cluster ‘overturned’ (in focus in panel C) for comparison (marked with asterisks *). ^1^: The fleur-de-lis is a stylized lily, used as a decorative design or symbol in the heraldry of Numerous European nations.

For each of the three meta-clusters (as separated by dotted lines in Figure 4B), the corresponding central structure is displayed in panels A, B, and C of Figure 5. In panel A, all three central structures of the corresponding meta-cluster (later to be named ‘open’) were shown in cyan. In addition to that *one* representative structure is shown out of the 17 frames, panel B focuses on (later to be named ‘closed’, red) and marked with an asterisk. Also, *one* representative frame is shown out of those five frames, panel C focuses on (later to be named ‘overturned’, green) and marked with an asterisk. The adjoining beta-sheets are displayed in grey, to facilitate a comparison to Figure 1.

Likewise, in panel B, all 17 central frames (6 blue, 6 red, 5 cyan) of the corresponding meta-cluster (later to be named ‘closed’) are shown. Again, for reference, we also showed representative central structures of the *two other* meta-clusters (later to be named ‘open’ in cyan, ‘overturned’ in green), marked with asterisks, including neighboring beta-sheets. Finally, panel C shows all 5 central frames of the third meta-cluster and reference information.

The visual inspection surprisingly revealed that within each meta-cluster (A, B, C) the central structures shared similar orientations of the CC′-loops. This meant that the unsupervised classification via MDS indeed extracted relevant structural features, readily confirmed by the visual inspection. What we found to be highly interesting is that each of these orientations of the CC′-loop has functional relevance and lends itself to name the meta-cluster accordingly.

Interestingly, the frames of two meta-clusters, panel (A) and panel (B), corresponded to conformations already known from previous experiments [15]: Zak described two conformations of the CC′-loop, ‘open’ and ‘closed’. We adopted this title and labeled the meta-clusters accordingly: panel A ≙ ‘open’ and in panel B ≙ ‘closed’ in Figure 5. In Figure 4 meta-clusters were also labelled accordingly.

Note that while the metaclusters emerged from an unsupervised procedure, the labels (‘open’ and ‘closed’) were only assigned after visual inspection and comparison with literature. For the sake of readability, we already used these labels in Figure 4 as well as in the text above.

**Figure 6:**
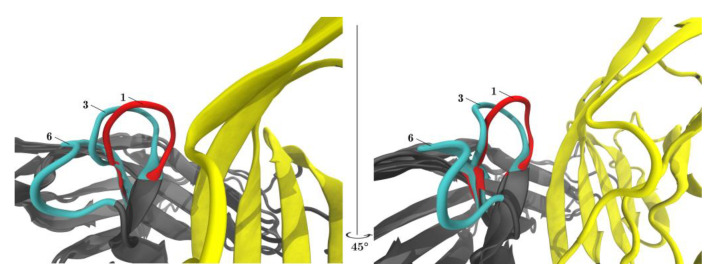
PD-L1 shifts the equilibrium of the CC′-loop towards the ‘closed’ conformation. The left and the right panel show the same content but rotated 45°. Superposition of the central structures of clusters 1, 3, (both members of meta-cluster ‘closed’) and 6 (meta-cluster ‘open’) are displayed with VMD. When PD-L1 binds to PD-1 (red) the CC′-loop is locked in the so-called ‘closed’ conformation (1) turned 90° towards PD-L1 (yellow). In the PD-1_Apo_ (cyan) the CC′-loop exhibits both the ‘open’ (6) and ‘closed’ conformation (3).

A closer look at the meta-clusters ‘open’ and ‘closed’, revealed that conformations switched back and forth within the same simulation (i.e., one and the same system), see Figure 6.

Figure 6 displayed both ‘open’ and ‘closed’ conformations within the same panel. Within one single simulation of PD-1_Apo_, ‘open’ and ‘closed’ conformations (e.g., central structures 6 and 3, cyan) were seen to coexist. The equilibrium between two conformations in PD-1_Apo_ also explained its increased RMSF in the CC′-loop, as seen in Figure 3, as well as the jump in RMSD, see Figure 2. Molecular dynamics results perfectly fit the experimental evidence, considering the switch between an ‘open’ and ‘closed’ conformation already described above [34].

In contrast to PD-1_Apo_, upon binding the natural ligand (PD-1_PD-L1_), the characteristic central structures (red) were found exclusively in the ‘closed’ metacluster, see Figure 4B. In fact, not only one but all conformations of PD-1_PD-L1_ had been assigned to clusters that fell within the meta-cluster ‘closed’ (Figure 4, compare panels A and B). This formal mathematical result was corroborated through visual inspection of Figure 5B, where all central structures of the CC′-loop originating from PD-1_PD-L1_ (red) appeared in the ‘closed’ position. We observed the following molecular mechanism in detail: While in the ‘open’ conformation the CC′-loop pointed away from the PD-L1 binding site, upon complex formation with PD-L1 the CC′-loop performed a 90° turn towards the ligand, allowing the formation of four hydrogen bonds between receptor and ligand (not shown), locking the CC′-loop in the ‘closed’ conformation. Our results clearly supported a shift in equilibrium from the ‘open’ to the ‘closed’ conformation upon PD-L1 binding (in agreement with the conformational selection model). This was in turn enabled by the rearrangement of the side chains of Ile134 (and Glu136), thereby making way for the CC′-loop [15]. Furthermore, Zak et al. described that the ‘closed’ CC′ conformation could also be found in murine PD-1_Apo_, leading to the hypothesis that the induced CC′-loop rearrangement was specific to human PD-1.

#### 3.2.1. Nivolumab Stabilizes ‘Closed’ Conformation

MDS also allocated all central structures originating from system PD-1_Niv_ to the ‘closed’ meta-cluster (Figure 4). Visual inspection (Panel B of Figure 5) revealed that these structures (blue) were indeed very similar to those originating from system PD-1_PD-L1_ (in red). We therefore concluded that Nivolumab also stabilized the ‘closed’ conformation of the CC′-loop.

#### 3.2.2. Pembrolizumab Induced a Novel CC′-loop Conformation

While nivolumab stabilized the ‘closed’ conformation, which was also accessible for PD-1_Apo_, pembrolizumab induced a new conformation which, to the best of our knowledge, has not been described to date and which we propose to call ‘overturned’. Figure 5 visualizes all three configurations and underlines naming the newly discovered one ‘overturned’ (Figure 5C):

Taking configuration ‘open’ (←) as reference, configuration ‘closed’ (↓) is twisted by about 90° (←↓). The new conformation (→) appears to be twisted by another 90° (↓→). Hence, relative to conformation ‘open’, the new conformation appears to be twisted by 180° (←→). Relative to the ‘open’ conformation, we call the ‘closed’ conformation ‘turned’ and the new conformation ‘overturned’, which is the name we will use to refer to this conformation henceforth.

This formally completes the naming of all three meta-clusters, including a rationalization for the used names. All central structures of this third meta-cluster originated from system PD-1_Pem_ (green), cf. the MDS result in Figure 4.

#### 3.2.3. Structural Switching, Binding Mechanisms and Clinical Relevance

Based on the visualization of the central frames (Figure 6) the ‘open’ and ‘closed’ conformation could be characterized (note the 90° turn towards the ligand in the ‘closed’ conformation) and marked accordingly in the distance map (Figure 4B). The meta-cluster of the ‘open’ conformation consisted only of structures from the PD-1_Apo_ simulation, which fit to the results of previous studies [15,16]. The meta-cluster with the ‘closed’ conformation accommodates structured of all four systems. The ratio of PD-1_Apo_ in ‘open’ and ‘closed’ conformation was 20 to 80. All structures of PD-1_PD−L1_ were in the ‘closed’ conformation. Our results supported the hypothesis that the binding of PD-L1 shifted the equilibrium of the CC′-loop towards the ‘closed’ conformation. Even though nivolumab did not bind to the CC′-loop it induced but in fact the ‘closed’ conformation. The results of this MD simulation showed that the CC′-loop of PD-1_Pem_ exhibited consecutively 85% of the time a conformation (Figure 5C) which had not been described in the literature to date. Upon pembrolizumab binding, the CC′-loop overturned by 180° relative to the ‘open’ conformation.

Although the ‘overturned’ configuration was characteristic for pembrolizumab, we saw these conformations also induced with nivolumab as ligand, to a minute degree: Clusters 10 and 17 (see panel A Figure 4), were mainly green (from PD-1_Pem_) but also contained a few configurations in blue (from PD-1_Niv_). This was important, because it indicated that this specific simulation could have gone into the overturned conformation if it had ‘wished’ to. Greatly prolonged simulation times will be needed to representatively sample such minute pockets of phase space.

Even though comparative binding analyses suggests that pembrolizumab binds PD-1 similar to PD-L1 [35], the involvement of the CC′-loop in the pembrolizumab binding process is still a matter of debate. This lack of clarity reflects the inconsistent data on the impact of PD-L1 expression on the efficacy of pembrolizumab in breast cancer. Horita et al. and Fessas et al. describe that the pembrolizumab binding site on PD-1 is composed of the CC′FG antiparallel β-sheet and the BC-, C’D- and FG-loops [36,37]. Lee et al. [38], on the other hand, mentioned the formation of a hydrogen bond between pembrolizumab and Thr76 which is part of the CC′-loop. Unfortunately, Lee et al. do not elaborate on the impact of the CC′-loop. Nevertheless, independent from the actual involvement of the CC′-loop in the pembrolizumab binding interface, the antibody does induce the ‘overturned’ conformation in the CC′-loop.

## 4. Conclusions

The aim of this study was to scrutinize a highly interesting molecule currently used clinically and therapeutically by methods of basic research in order to elucidate specific patterns of molecular motion. A thorough understanding of the binding dynamics could be used as the basis for improvements of therapeutics, focusing on the relevant characteristics of molecular interaction. A previous molecular dynamics simulation already focused on configurational dynamics and binding energies between PD-1 and its natural ligand [34]. Our own previous simulations covered the conformational impact on PD-1 upon binding to its natural ligand as well as to nivolumab [20]. The present work was an extension of that, also featuring pembrolizumab, and provided an interesting comparison between the different binding mechanisms of these two drugs.

### 4.1. Molecular Findings and Motivation

PD-1 is an immune checkpoint receptor which promotes apoptosis of antigen-specific T cells. Interaction with PD-L1 downregulates the activity of the adaptive immune system and prevents autoimmunity. The expression of PD-L1 is a common method for cancer cells to evade the immune system as it leads to apoptosis of tumor-specific T cells. Nivolumab and pembrolizumab, which belong to a new class of cancer drugs called PD-1 inhibitors, were developed more recently. Nivolumab and pembrolizumab are both monoclonal blocking antibodies which bind to PD-1 and shield it from the interaction with PD-L1. Predictive biomarkers are warranted to increase efficacy of immune checkpoint inhibition therapy. Especially regarding pembrolizumab, PD-L1 expression cannot be used as the only biomarker and a more in depth understanding of the mechanism of PD-1 is essential. The CC′-loop of PD-1 has previously been described as a hotspot for drug discovery [14].

We performed MD simulations of the complete extracellular domain of PD-1 in complex with PD-L1 and the blocking antibodies nivolumab and pembrolizumab. We were able to confirm that human PD-1_Apo_ displayed both the ‘open’ and the ‘closed’ conformation as described experimentally by Liu [16]. The experimental finding that CC′-loop was pivotal in the binding process was confirmed by our results, reflected in reduced motility (RMSF) upon ligand binding. The Daura et al. clustering algorithm and MDS were applied to classify the influence of the binding partners on the structure of the CC′-loop of PD-1. We were able to confirm that in non-ligated PD-1, the CC′-loop exhibited both the ‘open’ and ‘closed’ conformation in a ratio of 20% to 80%, respectively. Like PD-L1, nivolumab shifted the equilibrium towards the ‘closed’ conformation, in accordance with the conformational selection model. Pembrolizumab, on the other hand, induced a third conformation in the CC′-loop which had not been described to date. Upon pembrolizumab binding the CC′-loop turned by 180° (relative to configuration ‘open’) to form a novel conformation which we termed ‘overturned’. In short, configuration ‘open’ (←) was taken as reference, configuration ‘closed’ (↓) was termed ‘turned’ (by 90°) and the new configuration (→), being twisted by another 90°, was termed ‘overturned’ (by 180° = 90° + 90°). This insight will in future help with the optimization of already existing PD-1 antibodies and the future design of new drugs.

### 4.2. Methodological Advance

Regarding methodology, we further concluded that Daura et al. clustering and multidimensional scaling, although merely based on formal mathematical procedures, proved capable of meaningful discrimination of molecular structures that were uniquely different in function.

Upon pembrolizumab binding the CC′-loop formed the ‘closed’ conformation as well as a new conformation which has not been described to date. Pembrolizumab induced an overturning of the CC′-loop up to 180°. We termed this conformation ‘overturned’.

Confidence in our approach was increased by the fact that two essential conformations of the CC′-loop (‘open’ and ‘closed’) were discovered in an unsupervised procedure, i.e., only through the mathematical formalism (clustering and MDS), without any prior knowledge through literature or through experimentation. It is therefore clearly justified to assume that the third main configuration (‘overturned’) first described here, corresponds to reality. Daura et al. clustering followed by MDS may thus be considered a promising tool in the explanation of physiological findings in a molecular-mechanistic form or even in predicting new ones.

In the future we want to use unsupervised numerical methods to identify movement patterns throughout PD-1. Furthermore, we will consider performing simulations at a constant pH, since the preferred protonation state depends on the conformations, and these change over time.

## Supplementary Materials

The following are available online at https://www.mdpi.com/2072-6694/13/1/5/s1, Figure S1: Number of clusters increasing with total simulation time.
