# Supplementary material for: Pembrolizumab Induces an Unexpected Conformational Change in the CC′-loop of PD-1
Source: Cancers (Basel). 2020 Dec 22;13(1):5. doi: 10.3390/cancers13010005 (PMC7792774; doi:10.3390/cancers13010005)
Supplement: Supplementary file 1 [file cancers-13-00005-s001.pdf]

Supplementary material to Pembrolizumab induces an unexpected conformational change in the CC′-loop of PD-1

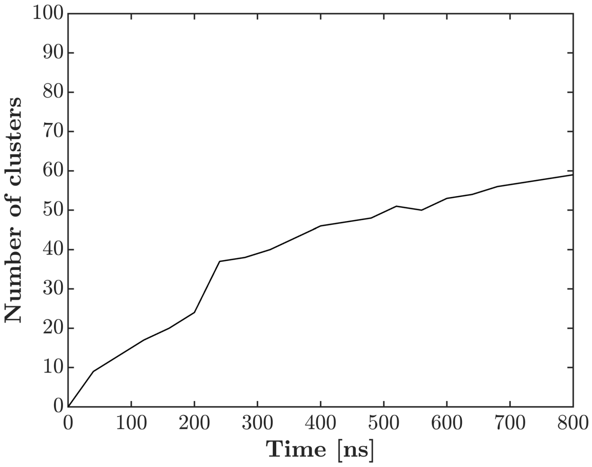

Figure S1. Number of clusters increasing with total simulation time. Daura clustering was performed over increasing portions (every 50 ns) of total simulation time (0-800 ns) of the four systems, PD-1$_{Apo}$, PD-1$_{PD\text{-}L1}$ (red), PD-1$_{Niv}$ (blue) and PD-1$_{Pem}$. First the number of clusters increases significantly and shows some levelling off later on, indicating that simulations have fairly converged.
